# Red blood cell distribution width predicts long term cardiovascular event after on-pump beating coronary artery bypass grafting

**DOI:** 10.1186/s13019-016-0465-4

**Published:** 2016-04-09

**Authors:** Orcun Gurbuz, Gencehan Kumtepe, Hakan Ozkan, Ilker Hasan Karal, Abdulkadir Ercan, Serdar Ener

**Affiliations:** Department of Cardiovascular Surgery, Balikesir University, School of Medicine, 10010 Balikesir, Turkey; Department Of Cardiology, Bahcesehir University Faculty of Medicine, Istanbul, Turkey; Department of Cardiovascular Surgery, Samsun Hospital for Education and Research, Ilkadim 55090 Samsun, Turkey; Department of Cardiovascular Surgery, Acıbadem Bursa Hospital, Bursa, Turkey

**Keywords:** Coronary artery bypass grafting, Major cardiovascular event, Red cell distribution width

## Abstract

**Background:**

Reports investigating the predictive value of red cell distribution width (RDW) on major cardiac and cardiovascular event (MACCE) following coronary artery bypass grafting (CABG) have major limitations, including lack of elimination of common factors affecting RDW levels, such as anemia. The purpose of this study is to identify the real effect of higher RDW level, free from the other factors, on MACCE following CABG.

**Methods:**

Data of 500 consecutive, non-anemic patients (77.2 % male and mean age 63.05 ± 9.24) undergoing ONBHCAB between January 2007 and January 2010, were analyzed retrospectively.

**Results:**

Overall MACCE was 7.8 % of all cases. Mean follow-up was 66.5 ± 9.96 months. In multivariate Cox regression analysis, RDW (*P* = 0.022) remained the only independent predictor of MACCE and the ROC analyze revealed an RDW cut-off value of 13.95 % predicting MACCE. Therefore, patients were grouped on this cut-off value. There were 238 patients in the lower RDW group (Group 1) and 262 patients in the higher RDW group (Group 2). Kaplan–Meier survival analysis of freedom from MACCE revealed significantly lower event free survival in Group 2 (*P* < 0.001 by the log-rank test). Group 2 showed a higher MACCE incidence in 1 year (*P* = 0.030), in 3 years (*P* < 0.001) and in 6 years (*P* < 0.001). The long-term follow-up was similar regarding noncardiovascular mortality.

**Conclusion:**

An RDW level greater than 13.95 % in hospital admission is independently associated with an increased incidence of MACCE after CABG*.* Physicians should be more aggressive in the management of these patients.

## Background

As the debate between coronary artery bypass grafting (CABG) and percutaneous coronary intervention (PCI) continues to increase, prediction of long-term survival following surgery has become critical [1, 2]. However, currently available risk assessment methods of predicting mortality following CABG have initially focused on the short term period [3, 4]. Therefore, knowing which patients should be closely monitored for cardiovascular events after surgery needs to be clarified.

The red blood cell distribution width (RDW) is a measurement of the size variation of erythrocytes used in the differential diagnosis of anemia [5]. Moreover, elevated RDW levels are shown to have a close relationship with the cardiovascular event in patients with coronary artery disease (CAD) [6, 7] or even in the general population [8]. Furthermore, a recent study also revealed a direct correlation between RDW level and the saphenous vein graft failure [9].

Several studies have shown a strong correlation between RDW level and cardiovascular event following percutaneous coronary intervention (PCI) [10, 11]. Unlike PCI, the medical literature contains relatively few reports investigating the role of RDW in the prediction of cardiovascular event following CABG [12, 13]. Moreover, these studies have some major limitations, including lack of elimination of factors affecting RDW level or not to be performed by a standard surgical technique. The primary endpoint of this study is to identify the pure effect of RDW on MACCE, defined as cardiac related or sudden death, ST elevation myocardial infarction (STEMI), the need for repeat revascularization and stroke following on-pump beating heart coronary artery bypass surgery (ONBHCAB) in a population free from factors affecting RDW level in hospital admission.

## Methods

### Study population

The study population consisted of 1044 patients who underwent elective isolated coronary bypass surgery at Bursa Medical Park Hospital between January 2007 and January 2010. The research was conducted according to the principles of the Declaration of Helsinki.

Exclusion criteria were as follows: critical preoperative state (need for inotropic drug support or intra-aortic balloon pumping (IABP), acute renal failure, requiring respiratory support, history of cardiopulmonary resuscitation in the preoperative period), myocardial infarction (MI) within 3 weeks (cTnI > 0.01 ng/ml), anemia (13 g/dL hemoglobin in men and 12 g/dL hemoglobin in women), hepatic dysfunction (alanine transaminase more than twice the upper limit of normal), renal dysfunction (creatinine >1.5 mg/dl) or thyroid dysfunction, thrombocytopenia (thrombocyte level <150,000/uL) or thrombocytosis (thrombocyte level >400,000/uL), leukocytosis (white blood cell level >11,000 /uL) or active infection and missing data.

Three hundred and thirty three patients were excluded from the study because they had anemia at admission; 56 patients were excluded due to renal dysfunction; 22 patients were excluded due to MI within 3 weeks; 4 patients were excluded because of thyroid dysfunction; 18 patients were excluded because of thrombocytosis or thrombocytopenia, 23 patients were excluded due to leukocytosis or active infection and 88 patients were excluded due to missing preoperative RDW level or long-term outcome data. Finally, 500 patients (77.2 % male and mean age 63.05 ± 9.24) were included in this retrospective study.

### Definitions

Patients’ preoperative characteristics, such as age and sex, smoking status, hypertension, diabetes mellitus (DM), hyperlipidemia, family history of CAD, obesity (the body mass index >30 kg/m^2^), chronic obstructive pulmonary disease (COPD), history of stroke, peripheral vascular disease (PVD), asymptomatic carotid stenosis, history of myocardial infarction (MI), unstable angina pectoris (USAP)**,** EuroSCORE II **(**European System for Cardiac Operative Risk Evaluation) risk score, left ventricular dysfunction, mitral insufficiency, history of PCI, the number of vessel disease, the presence of left main coronary artery (LMCA) stenosis were recorded.

The diagnosis of DM was based on previous history of diabetes or fasting plasma glucose ≥126 mg/dl or hemoglobin A1C ≥6.5 %. The diagnosis of hyperlipidemia was based on previous history or total cholesterol ≥200 mg/dl or LDL ≥130. Anemia was defined as a baseline hemoglobin (Hb) concentration less than 13 mg/dl in men and less than 12 mg/dl in women, in accordance with the World Health Organization criteria. Vessel disease was defined by a stenosis of >50 % of major epicardial coronary arteries. Estimated creatinine clearance (CrCl) was calculated using the Cockcroft-Gault formula: CrCl (ml/min) = ([140-age] x weight [kg]) /(serum creatinine [mg/dl] x 72) (x 0.85 for women) from baseline blood samples. The diagnosis of COPD was based on previous history of bronchodilatator treatment or the FEV1/FVC ratio <0.70. Asymptomatic carotid stenosis was defined as Doppler duplex sonography revealed a greater than 50 % in the internal carotid artery. PVD was defined as arterial disease affecting the non-carotid vasculature. The left ventricular dysfunction was defined moderate (EF, 0.30 to 0.49) or severe (EF <0.30).

Preoperative and postoperative laboratory tests and outcomes were retrospectively collected from the hospital records. Complete revascularization was defined as treatment of all major coronary arteries ≥50 % diameter stenosis. Number of concomitant endarterectomy per anastomosis was calculated. Drainage was defined as the sum of the mediastinal and chest tubes’ drainage in the first 24 h. Consumed unit of blood was defined as the sum of the blood unit used during the hospital stay. Any inotropic support started in the perioperative period, even low-dose of dopamine infusion, was determined as the perioperative need for inotropic support. Perioperative non-ST elevation MI (NSTEMI) was defined as cardiac Troponin I (cTnI) >5 μg/L during the hospital stay without new electrocardiography ECG change [14]. Perioperative STEMI was defined as cardiac Troponin I (cTnI) >5 μg/L during the hospital stay with new ECG change or echocardiography evidence of new regional wall motion abnormality. Postoperative renal failure was defined as an increase ≥100 % in basal serum creatinine. Pulmonary complication was defined as pleural effusion, atelectasis, phrenic nerve paralysis, diaphragmatic dysfunction, pneumonia, acute respiratory distress syndrome, pneumothorax or chylothorax. Neurologic complication was defined as any new TIA, stroke or encephalopathy occurring in the perioperative period. Early reoperation was defined as any hospitalization due to CABG-related complications (such as sternal dehiscence, mediastinitis) or cardiovascular problems (such as MI, congestive heart failure, rhythm disturbance, neurologic complications, pulmonary embolism).

Long-term follow-up was obtained through clinic visits, hospital records and phone calls. All-cause mortality (patient death reported by patients’ relatives or hospital records) and MACCE (STEMI, repeat coronary revascularization (repeat CABG or PCI), stroke, cardiac related or sudden death) was determined.

### Surgical procedures

All procedures were performed by the same surgeon or under his supervision using the ONBHCAB technique. Classic median sternotomy, left internal thoracic artery (LIMA) harvesting and other conduits’ preparations were performed by a standard technique. Heparin was administered to keep the activated clotting time (ACT) greater than 450 s during surgery. All procedures were performed without using an aortic cross-clamping and cardioplegia. Cardiopulmonary bypass (CPB) was established with an ascending aortic arterial cannula and a right atrial two stage venous cannula, using a membrane oxygenator and a roller pump. All patients were cooled to 32–34 °C. Mean arterial blood pressure was maintained in the range of 60–90 mmHg. Distal anastomoses were performed by end-to-side or side-to-side techniques with a running 7/0 prolene suture, using a myocardial stabilizer device (Octopus IV, Medtronic Inc., Minneapolis, MN, US). Proximal anastomoses were performed using 6/0 prolene suture during the heating period with the assistance of ascending aortic side-clamp. After the completion of CPB and cannula removal, heparin was neutralized with protamine providing an ACT less than 160 s. Acetylsalicylic acid at a dose of 100 mg and low molecular-weight heparin was initiated on the postoperative 24th hours. All the patient was discharged under acetylsalicylic acid therapy.

### Laboratory analysis and echocardiography

In all patients, RDW and all other hematological indices were measured, as part of the automated complete blood count (CBC), using a Cell-Dyn 3700 Hematology Analyzer (Abbott Diagnostics, Santa Clara, CA, USA). The reference range for RDW was 10–15 %. Biochemical analyzes were performed with the Architect ci8200 Chemistry Analyzer (Abbott Diagnostics, Santa Clara, CA, USA).

Transthoracic echocardiography was performed for each patient before surgery using a Vivid S3 (GE Healthcare, Milwaukee, WI, USA) with a 1.5–3.6 MHz phased array transducer. The left ventricular ejection fraction (LVEF) was measured using the modified Simpson’s rule [15].

### Clinical endpoints

The primary endpoint of this study is to identify the effect of RDW on early and late MACCE, defined as cardiac related or sudden death**,** STEMI, the need for repeat revascularization and stroke following CABG in patients who have not any factor affecting RDW level in hospital admission. The secondary endpoints were to identify independent predictors of MACCE in patient undergoing ONBHCAB and the effect of RDW on in-hospital morbidity.

### Statistical analysis

Continuous variables were expressed as mean ± standard deviation. Categorical variables are expressed as percentages. The Mann–Whitney U test was used to compare nonparametric continuous variables, the Student’s t-test was used to compare parametric continuous variables and the chi-square test was used to compare categorical variables. The cumulative survival curves for long term MACCE was constructed with the use of the Kaplan-Meier method, whereas differences between the RDW groups were evaluated with log-rank tests. The receiver operating characteristics (ROC) curve was used to detect optimal cut-off value for predicting MACCE. Cox regression analysis was performed to determine independent predictors of MACCE, with those variables with a *P*-value of <0.05 in the univariate analysis being included in the stepwise multivariate model. The hazard ratio (HR) and 95 % confidence intervals (CI) were calculated. The association between variables was tested using Spearman or Pearson’s correlation coefficient. Two-tailed *p* values < 0.05 were considered as significant. All statistical analyses were conducted out using the Statistical Package for Social Sciences (SPSS) program (version 15.0, SPSS, Chicago, Illinois, USA).

## Results

The variables for which the *p* value was <0.05 in univariate Cox analysis (age, EuroScore II, hypertension, preoperative RDW value, asymptomatic carotid artery stenosis, average number of vessel disease, three vessel disease, family history of CAD, previous MI, duration of hospital stay, duration of ICU stay, prolonged respiratory period, the mean number of red blood cell transfusion units, rehospitalization) were identified as potential risk factors for MACCE (Table 1) and these variables were analyzed with multivariate Cox regression model. In multivariate Cox regression analyses, RDW (hazard ratio (HR) 1.227, <95 % CI 1.052–1.430; *P* = 0.009) remained the only independent predictor of MACCE following ONPBHCAB (Table 1).

**Table 1:** Effects of multiple variables on the MACCE in Cox Regression analysis

	Univariate Analysis			Multivariate Analysis		
Characteristics	HR	95 % CI	*P*	HR	95 % CI	*P*
Age (years)	1.063	1.026–1.102	0.001*	1.024	0.974–1.076	0.36
Male Sex	1.038	0.491–2.192	0.92			
Obesity	1.444	0.701–2.977	0.31			
CrCL	0.99	0.978–1.001	0.08			
hs-CRP	1.003	0.994–1.013	0.51			
RDW	1.175	1.044–1.322	0.007*	1.227	1.052–1.430	0.009*
Hg	0.755	0.472–1.209	0.24			
EuroSCORE II	1.099	1.043–1.159	<0.001*	1.069	0.955–1.1197	0.24
USAP	0.941	0.457–1.937	0.87			
History of MI (>21 days)	1.952	1.017–3.749	0.044*	2.135	0.934–4.883	0.07
History of PCI-CABG	0.496	0.123–2.007	0.32			
Current smoker	0.630	0.322–1.232	0.17			
Family history of CAD	0.399	0.175–0.905	0.028*	0.450	0.185–1.091	0.07
Diabetes mellitus	1.155	0.591–2.259	0.67			
Hyperlipidemia	0.928	0.451–1.911	0.84			
Hypertension	2.391	1.206–4.738	0.013*	2.064	0.967–4.404	0.06
COPD	1.630	0.578–4.594	0.35			
Asymtomatic Carotid Stenosis	2.206	1.101–4.420	0.026*	1.353	0.579–3.159	0.48
PVD	0.947	0.242–3.708	0.93			
History of Stroke	1.131	0.155–8.260	0.90			
LMCA stenosis	1.957	0.896–4.272	0.09			
Average number of vessel disease	2.238	1.081–4.633	0.030*	1.275	0.162–10.031	0.81
Three vessel disease	2.728	1.141–6.524	0.024*	1.664	0.126–22.034	0.69
Severe left ventricular dysfunction	1.481	0.455–4.821	0.51			
Mitral insufficiency	1.873	0.824–4.253	0.13			
Number of distal anastomosis	1.396	0.998–1.953	0.05			
Endarterectomy per anastomosis	1.358	0.715–2.577	0.35			
Duration of hospital stay	1.143	1.072–1.218	<0.001*	1.070	0.935–1.224	0.32
Prolonged respiratory period	3.673	2.109–6.398	<0.001*	2.417	0.466–12.551	0.29
Duration of ICU stay	1.020	1.009–1.032	<0.001*	1.002	0.969–1.035	0.92
Perioperative AF	2.026	0.926–4.421	0.07			
Perioperative MI	0.993	0.352–2.804	0.99			
Pulmonary Complication	1.451	0.793–2.655	0.22			
Perioperative Renal Failure	1.880	0.890–3.973	0.09			
Cerebrovascular Complication	1.465	0.519–4.130	0.47			
Mean number of blood unit	1.204	1.008–1.438	0.041*	0.851	0.618–1.197	0.24
Rehospitalization	1.789	1.014–3.157	0.045*	1.823	0.925–3.592	0.08

*Statistically significant difference. Values are presented as mean ± standard deviation or number (%), where appropriate. *AF* atrial fibrillation, *CAD* coronary artery disease, *CI* confidence interval**,** *COPD* chronic obstructive pulmonary disease, *CrCL* creatinin clearance, *hs-CRP* high sensitive C-reactive protein, *EuroSCORE II* European System for Cardiac Operative Risk Evaluation, *Hg* haemoglobin level, *HR* hazard ration, *ICU* intensive care unit, *LMCA* left main coronary artery, *MACCE* major cardiac and cerebrovascular event, *MI* myocardial infarction, *MPV* mean platelet volume, *OR* odds ratio**,** *PCI* percutaneous coronary intervention, *PVD* peripheral vascular disease, *RDW* red blood cell distribution width, *USAP* unstabil angina pectoris

**Fig. 1:**
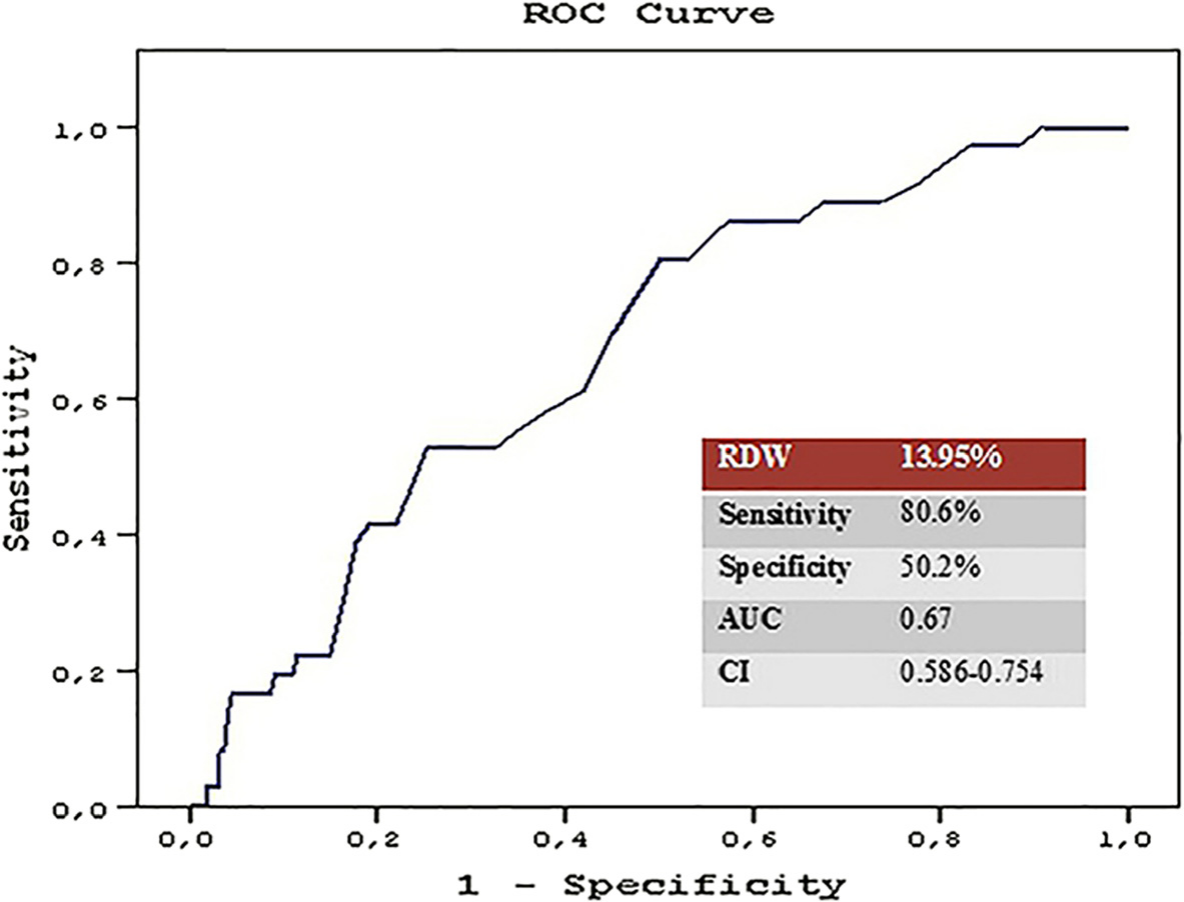
The receiver-operating characteristic (ROC) curve of RDW for predicting MACCE (RDW: red cell distribution width, AUC: area under curve, CI: confidence interval)

The ROC curves of RDW revealed that an RDW >13.95 % measured on admission had 80.6 % sensitivity and 50.2 % specificity in predicting MACCE after ONBHCAB (Fig. 1). Therefore, we divided the study population into two groups according to this cut-off value.

After the evaluation of the patients’ data according to our inclusion and exclusion criteria, 500 patients were included in this study. There were 238 patients (mean age 60.76 ± 9.3 and 80.3 % male) in the lower RDW group (Group 1) and 262 patients (mean age 65.13 ± 8.68 and 74.4 % male) in the higher RDW group (Group 2). Baseline characteristics are shown in Table 2. The preoperative characteristics of the groups were similar regarding sex, obesity, hypertension, hyperlipidemia, family history of CAD, COPD, DM, history of stroke, peripheral vascular disease, asymptomatic carotid stenosis, history of MI, moderate and severe LV dysfunction, USAP, previous PCI, LMCA stenosis and three vessel diseases. With respect to baseline laboratory status, the RDW levels on admission were significantly higher (*P* < 0.001) and hemoglobin level was significantly lower (*P* < 0.001) in Group 2, while there was no significant difference in high sensitive C-reactive protein level (hs-CRP) between groups.

**Table 2:** Baseline demographic and clinical parameters according to RDW levels

Characteristics	Overall *n* = 500	RDW < 13.95 *n* = 238 (47.6)	RDW > 13.95 *n* = 262 (52.4)	*p*-value
Age (years)	63.05 ± 9.24	60.76 ± 9.3	65.13 ± 8.68	<0.001*
Male	386 (77.2)	191 (80.3 %)	195 (74.4)	0.12
Hg	13.19 ± 0.65	13.31 ± 0.62	13.07 ± 0.65	<0.001*
RDW	14.32 ± 1.72	13.14 ± 0.53	15.39 ± 1.74	<0.001*
MPV	10.08 ± 1.15	9.93 ± 1.29	10.21 ± 1	0.012*
hs-CRP	15.77 ± 29.3	13.34 ± 21.59	17.96 ± 34.74	0.17
CrCL	99.7 ± 32.98	105.07 ± 34.08	94.36 ± 31.03	0.001*
EuroScore II	3.46 ± 3.26	3.02 ± 3.07	3.87 ± 3.38	<0.001*
Obesity (BMI ≥ 30)	134 (26.8)	62 (26.1)	72 (27.5)	0.38
Current Smoker	221 (44.2)	117 (49.2)	104 (39.7)	0.033*
Hypertension	240 (48)	109 (45.8)	131 (50)	0.34
Hyperlipidemia	137 (27.4)	62 (26.1)	75 (28.6)	0.52
Family history of CAD	174 (34.8)	90 (37.8)	84 (32.1)	0.17
Diabetes Mellitus	156 (31.2)	70 (29.4)	86 (32.8)	0.41
COPD	34 (6.8)	13 (5.5)	21 (8)	0.25
History of Stroke	13 (2.6)	4 (1.7)	9 (3.4)	0.22
PVD	25 (5)	8 (3.3)	17 (6.5)	0.11
Asymtomatic Carotid Stenosis	86 (18.2)	32 (14.5)	54 (21.5)	0.048*
History of MI	125 (25)	59 (24.8)	66 (25.2)	0.92
Moderate LV dysfunction	27 (135)	64 (26.9)	71 (27.1)	0.95
Severe LV dysfunction	29 (5.8)	12 (5)	17 (6.5)	0.63
Mild mitral insufficiency	55 (11)	17 (7.1)	38 (14.5)	0.009*
USAP	139 (27.8)	68 (28.6)	71 (27.1)	0.71
Previous PCI	46 (9.2)	20 (8.4)	26 (9.9)	0.55
Number of vessel disease	2.58 ± 0.65	2.51 ± 0.7	2.65 ± 0.59	0.032*
LMCA stenosis	83 (16.6)	37 (15.5)	46 (17.6)	0.50
Three Vessel disease	336 (67.2)	150 (63)	186 (71)	0.06

*Statistically significant difference. Values are presented as mean ± standard deviation or number (%), where appropriate. *CAD* coronary artery disease, *CrCL* creatinin clearance, *COPD* chronic obstructive pulmonary disease, *Hb* haemoglobin, *hs-CRP* C-reactive protein, *EuroSCORE II* European System for Cardiac Operative Risk Evaluation, *DM* diabetes mellitus, *LMCA* left main coronary artery, *LV* left ventricule, *MI* myocardial infarction, *MPV* mean platelet volume, *n* number, *PCI* percutaneous coronary intervention, *PVD* peripheral vascular disease, *RDW* red blood cell distribution width, *TIA* transient ischemic attack, *USAP* unstabil angina pectoris

The patients with higher RDW values were significantly older (Group 1, 60.76 ± 9.3 years; Group 2, 65.13 ± 8.68; *P* < 0.001) or had significantly higher mean EuroScore II (Group 1, 3.02 ± 3.07; Group II, 3.87 ± 3.38; *P* < 0.001), higher mean number of vessel disease (Group 1, 2.51 ± 0.7 years; Group 2, 2.65 ± 0.59; *P* = 0.032) or more mild mitral insufficiency (Group 1, 17 (7.1 %); Group 2, 38 (14.5 %); *P* = 0.009) or lower mean CrCL (Group 1, 105.07 ± 34.08; Group 2, 94.36 ± 31.03; *P* = 0.001). The patients with lower RDW had a significantly higher current smoker (Group 1, 117 (49.2 %); Group 2, 104 (39.7 %); *P* = 0.033).

Peri-operative and early post-operative patients’ characteristics are shown in Table 3. The perioperative and early postoperative characteristics of the two groups were similar regarding complete revascularization, mean number of grafted LAD or CX or RCA, early re-operation, early rehospitalization, stroke, pulmonary complication, perioperative MI, perioperative AF, mediastinitis and early mortality or MACCE. The patients with higher RDW values showed significantly more mean distal anastomosis per patient (Group 1, 3.31 ± 0.4; Group 2, 3.53 ± 0.87; *P* = 0.011), longer duration of hospital stay (Group 1, 5.5 ± 1.98; Group 2, 6.12 ± 2.54; *P* < 0.001), more postoperative drainage (Group 1, 487.6 ± 313 ml/24 h, Group 2, 537.7 ± 293 ml/24 h; *P* = 0.012), higher need of blood transfusion (Group 1, 0.9 ± 1.4 unit; Group 2, 1,64 ± 1.48 unit; *P* < 0.001), higher need of inotropic agent (Group 1, 7 (2.9); Group 2, 22 (8.4 %), *P* = 0.009), more neurological complication (Group 1, 6 (2.5 %); Group 2; 33 (12.6 %); *P* < 0.001), more encephalopathy (Group1, 2 (0.8 %); Group 2, 11 (4.2 %); *P* = 0.019) or TİA (Group 1, 4 (1.7); Group 2, 20 (7.6 %); *P* = 0.002) and more perioperative renal failure (Group 1, 20 (8.4); Group 2, 54 (20.6); *P* < 0.001).

**Table 3:** Perioperative and early postoperative characteristics of the patients stratified by RDW levels

Characteristics	Overall *n* = 500	RDW < 13.95 *n* = 238 (47.6)	RDW > 13.95 *n* = 262 (52.4)	*p*
Number of distal anastomoses	3.43 ± 0.93	3.31 ± 0.4	3.53 ± 0.87	0.011*
Complete Revascularization	486 (97.2)	230 (96.6)	256 (97.7)	0.47
Number of grafted LAD	1.67 ± 0.57	1.64 ± 0.56	1.7 ± 0.58	0.28
Number of grafted Cx	0.97 ± 0.65	0.93 ± 0.65	1.01 ± 0.66	0.10
Number of grafted RCA	0.77 ± 0.53	0.74 ± 0.54	0.79 ± 0.52	0.23
Endarterectomy (per anastomosis)	64 (3.72)	28 (3.54)	36 (3.88)	0.43
Duration of hospital stay (days)	5.82 ± 2.31	5.5 ± 1.98	6.12 ± 2.54	<0.001*
Prolonged respiratory period (>12 h)	3 (0.6)	1 (0.4)	2 (0.8)	0.62
Duration of intensive care unit (h)	20.62 ± 14	20.92 ± 17.9	20.34 ± 9.17	0.40
Drainage (ml/24 h)	513.9 ± 303.5	487.6 ± 313	537.7 ± 293,4	0.012*
Blood transfusion (unit)	1.29 ± 1.49	0.9 ± 1.4	1,64 ± 1.48	<0.001*
Perioperative need for inotropic support	29 (5.8)	7 (2.9)	22 (8.4)	0.009*
Perioperative need for IABP	3 (0.6)	3 (1.3)	1 (0.4)	0.27
Perioperative NSTEMI	57 (11.4)	27 (11.3)	30 (11.5)	0.75
Perioperative AF	62 (12.4)	30 (12.6)	32 (12.2)	0.89
Perioperative Renal failure	74 (14.8)	20 (8.4)	54 (20.6)	<0.001*
Pulmonary complication	9 (1.8)	3 (1.3)	6 (2.3)	0.38
Neurological complication	39 (7.8)	6 (2.5)	33 (12.6)	<0.001*
Encephalopathy	13 (2.6)	2 (0.8)	11 (4.2)	0.019*
TIA	24 (4.8)	4 (1.7)	20 (7.6)	0.002*
Stroke	2 (0.4)	0	2 (0.8)	0.17
Mediastinitis	3 (0.6)	2 (0.8)	1 (0.4)	0.50
Early Re-operation	13 (2.6)	3 (1.2)	10 (3.8)	0.07
Early Re-operation due to bleeding	9 (1.8)	2 (0.8)	7 (2.7)	0.12
Early Rehospitalisation (<30 days)	45 (9)	17 (7.1)	28 (10.7)	0.32
Mortality (<30 days)	2 (0.4)	1 (0.4)	1 (0.4)	0.94
MACCE (<30 days)	4 (0.8)	1 (0.4)	3 (1.2)	0.36

*Statistically significant difference. Values are presented as mean ± standard deviation or number (%), where appropriate. *AF* Atrial fibrillation, *Cx* circumflex coronary artery, *FFP* fresh frozen plasma, *IABP* intra aortic balloon pump, *LAD* left anterior descending coronary artery, *LIMA* left internal mammary artery, *MACCE* major cardiac and cerebrovascular event, *MI* myocardial infarction, *NSTEMI* non-ST elevation MI, *RCA* right coronary artery, *RDW* red blood cell distribution width, *RİMA* right internal mammary artery, *TIA* transient ischemic attack

**Fig. 2:**
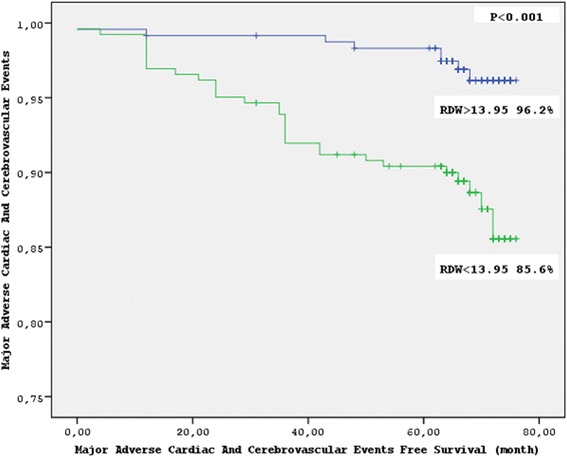
Kaplan–Meier Estimates of Free Survival from Cardiovascular Death, Stroke, Myocardial Infarction and Repeated Coronary Revascularization (*P* < 0.001 by the log-rank test)

Six-year follow-up characteristics of patients are shown in Table 4. The higher RDW group showed a significantly lower MACCE free survival (Group 1, 67.68 ± 8.54 months; Group 2, 63.19 ± 14.76 months; *P* < 0.001), higher MACCE in 1 year (Group 1, 2 (0.8 %); Group 2, 10 (3.8 %); *P* = 0.030), in 3 years (Group 1, 2 (0.8 %); Group 2, 21 (8 %); *P* < 0.001) and in 6 years (Group 1, 8 (3.4 %); Group 2, 31 (11.8 %); *P* < 0.001). Moreover, patients with higher RDW values showed higher all-cause mortality in 6 years (Group 1, 7 (2.9 %); Group 2, 22 (8.4 %); *P* = 0.009), more STEMI (Group 1, 1 (0.4 %); Group 2, 7 (2.7 %); *P* = 0.045) and TIA (Group 1, 5 (2.1 %); Group 2, 24 (9.2 %); *P* = 0.001). The long term follow-up of the two groups was similar regarding stroke, late reintervention and noncardiovascular mortality. Accordingly, Kaplan–Meier analysis of freedom from MACCE revealed significantly lower event-free survival in the higher RDW group (Group 1, 96.6 %; Group 2, 85.6 %; *P* < 0.001 by the log-rank test) (Fig. 2).

**Table 4:** Long-term outcomes of the patients, according to RDW levels

Characteristics	Overall *N* = 500	RDW < 13.95 *n* = 238 (47.6)	RDW > 13.95 *n* = 262 (52.4)	*p*
Mean follow*-*up time (mounth)	66.5 ± 9.96	67.69 ± 8.54	65.43 ± 11	0.004*
MACCE Free Survival	65.33 ± 12.4	67.68 ± 8.54	63.19 ± 14.76	<0.001*
MACCE (1 year)	12 (2.4)	2 (0.8)	10 (3.8)	0.030*
MACCE (3 year)	23 (4.6)	2 (0.8)	21 (8)	<0.001*
MACCE (6 year)	39 (7.8)	8 (3.4)	31 (11.8)	<0.001*
STEMI	8 (1.6)	1 (0.4)	7 (2.7)	0.045*
Total Stroke	9 (1.8)	2 (0.8)	7 (2.7)	0.12
Total TIA	29 (5.8)	5 (2.1)	24 (9.2)	0.001*
Late reintervention	13 (2.6)	4 (1.7)	9 (3.4)	0.22
Stent	12 (2.4)	4 (1.7)	8 (3.1)	0.31
Redo-CABG	1 (0.2)	0	1 (0.4)	0.34
All-cause mortality	29 (5.8)	7 (2.9)	22 (8.4)	0.009*
Cardiovascular mortality	19 (3.8)	4 (1.7)	15 (5.3)	0.018*
Non-Cardiovascular mortality	10 (2)	3 (1.3)	7 (2.7)	0.26

*Statistically significant difference. Values are represented as mean ± standard deviation and number (%), where appropriate. *MACCE* major cardiac and cerebrovascular event, *STEMI* ST elevation MI, *TIA* Transient ischemic attack

## Discussion

In this study, we showed for the first time that baseline RDW levels were independently associated with mid and long-term, but not short-term, MACCE following CABG especially in a non-anemic population. The strengths of our analysis include elimination of common factors affecting RDW levels, the use of a single hematology analyzer for all assays and also the use of a standard surgical technique.

Despite, the traditional usage of RDW has been restricted to differential diagnosis of anemia, [5, 16] elevated RDW levels were found to be strongly correlated with cardiovascular event following PCI [7, 10] or acute MI [7] and also in patients with heart failures [17] or in the general population [18]. Furthermore, situations which have prognostic importance in cardiovascular disease such as decreased kidney function, [19] SYTAX score, [20] carotid stenosis [21] were found to be closely associated with increased RDW levels. RDW reflects the anisocytosis which is caused by both impaired erythropoiesis and abnormal red blood cell survival due to a variety of underlying metabolic abnormalities such as oxidative stress, inflammation, poor nutritional status, hyperlipidemia, hypertension, erythrocyte fragmentation and alteration of erythropoietin function [22]. Therefore, the mechanism underlying increased RDW level, especially in non-anemic population seems to be closely related with cardiovascular risk factors.

Although the prognostic value of RDW in cardiovascular events following PCI are well known, [7, 10, 11, 23, 24] the effect of RDW on MACCE in the CABG patients has not been adequately evaluated [12, 13]. We revealed that the increased RDW level is associated factors affecting severity and complexity of atherosclerosis such as, increased age, higher mean number of vessel disease, higher asymptomatic carotid stenosis and lower circle, similar to previous studies [19–22]. Moreover, hypertension, age, three vessel disease, asymptomatic carotid stenosis were found to be predictors of MACCE following CABG in univariate analysis. Benedetto U [12] et al. showed that higher RDW levels are associated with increased CRP level as an inflammatory marker in CABG patients. However, in contrast to previous studies we have not shown any correlation with RDW and CRP level [12, 22]. This finding might be related to the exclusion of patients with active infection and leukocytosis, unlike previous studies. This suggests that inflammation is not the major mechanism for the increased incidence of MACCE in the present study.

Correlated to report of Warwick et al., [13] we also revealed that higher RDW levels were related to significantly more postoperative drainage and need for blood units. However, in their study anemia limit was 11 g/dl for both genders, which might affect their finding. It is clear that the blood damage is an unavoidable side effect of extracorporeal circulation, because of this the requirement of erythropoiesis increases following on-pump CABG. Higher levels of RDW within the normal range indicate accelerated red blood cell destruction or, more commonly, ineffective erythropoiesis [5]. Therefore, increased need for blood transfusion might be explained by the combined effect of CPB and impaired erythropoiesis. Further studies are needed to evaluate the impact of increased RDW on blood transfusion in patient undergoing off pump CABG (OPCAB).

A previous study showed that higher RDW levels are related to increased reperfusion injury following PCI [25]. Accordingly, we revealed that higher RDW levels are associated with the more inotropic support need even similar baseline LV dysfunction or perioperative cTnI levels between groups. As all the patients have operated under the supervision of one experienced surgeon using ONPBH technique. This temporary postoperative ventricular dysfunction and the need for inotropic support might be explained that higher RDW levels might be associated with increased reperfusion injury frequency due to microvascular dysfunction or non-reflow phenomenon in patients undergoing coronary bypass surgery.

As preoperative CrCL levels were significantly lower, preoperative renal failure was significantly more detected in the higher RDW group. Even though patients with abnormal creatine level were excluded from our study, the higher RDW level might be a predictor of hidden renal failure, as Lippi G. et al. [19] reported. Moreover, perioperative encephalopathy and TIA are significantly more detected correlated with more preoperative asymptomatic carotid stenosis in the higher RDW group. However, no difference detected between groups in terms of perioperative stroke.

Unlike the previous studies in CABG patients, higher RDW levels were found not to be associated with in hospital mortality or early MACCE following CABG [12, 13]. However, these findings might be affected by the relatively small sample size. Correlated with the previous publications the patients with higher RDW levels showed a significantly lower MACCE free survival, and also higher MACCE in 1 year, in 3 years and in 6 years. Furthermore, we found that the non-cardiovascular mortality is not associated with higher RDW levels in CABG patients, unlike cardiovascular mortality. Therefore, the higher all-cause mortality seems to be mainly affected by cardiovascular mortality. As many chronic and inflammatory diseases cause anemia, elimination of anemic patients might decrease the non-cardiovascular mortality. A recent study conducted on non-anemic patients with a history of CABG revealed a direct correlation between RDW level and the saphenous vein graft failure and similar to our finding the mean increased RDW level was detected between the normal range [9]. Further studies are needed to elucidate the mechanism of RDW impact on long-term MACCE in patient undergoing CABG.

Possible limitations of the present study are: First, the study was conducted in a single center and included a relatively small number of patients. However, our population contains homogeneous, non-anemic CABG patients submitted to ONPBH under the same experienced surgeon supervision; therefore, the factors which interact with RDW level or frequency of MACCE due to differences in surgical technique were excluded. Second, excluding anemic patients and patients with renal or hepatic or thyroid dysfunction caused to eliminate approximately half of the population, which caused a significant reduction of the sample size. However, this elimination gave us the opportunity to realize real relationship between RDW levels and MACCE in patient undergoing CABG, unlike the previous studies [12, 13]. Third, the single center nature also ensured that all the blood samples were studied with the same hematology analyzer securing the reference value changes.

## Conclusion

An RDW level greater than 13.95 % is independently associated with risk of MACCE following coronary bypass surgery in the long term. Moreover, it also predicts increased perioperative morbidity. We believe that RDW level, which is a simple tool routinely used in daily clinical practice as a part of the CBC, might be used as a predictor of MACCE and patient with higher RDW supposed to be under more frequent control against cardiovascular events. As we eliminated other factors increasing RDW level, 13.95 might be accepted as a threshold for cardiovascular events. Therefore, further studies with larger sample size are needed to determinate a certain cut-off value for RDW level and to test the effect of RDW on OPCAB surgery.

## Abbreviations

ACT: activated clotting time
AF: atrial fibrillation
CABG: coronary artery bypass graft
CAD: coronary artery disease
COPD: chronic obstructive pulmonary disease
CPB: cardiopulmonary bypass
CrCl: creatinine clearance
cTnI: cardiac Troponin I
Cx: circumflex coronary artery
DM: diabetes mellitus
ECG: electrocardiography
EF: ejection fraction
EuroSCORE II: European System for Cardiac Operative Risk Evaluation
FEV1: forced expiratory volume in 1 second
FVC: the forced vital capacity
Hb: hemoglobin
IABP: İntra-aortic balloon pumping
ICU: intensive care unit
LAD: left anterior descending artery
LIMA: left internal mammary artery
LMCA: left main coronary artery
MACCE: major adverse cardiac and cerebrovascular events
MI: myocardial infarction
NSTEMI: non-ST elevation MI
ONBHCAB: on-pump beating-heart CABG
OR: odds ratio
PCI: percutaneous cardiac intervention
PVD: peripheral vascular disease
RCA: right coronary artery
RDW: red blood cell distribution width
RIMA: right internal mammary artery
ROC: receiver operating characteric
STEMI: ST elevation MI
TIA: transient ischemic attack
